# Native T1 Mapping in Assessing Kidney Fibrosis for Patients With Chronic Glomerulonephritis

**DOI:** 10.3389/fmed.2021.772326

**Published:** 2021-10-18

**Authors:** Jianhua Wu, Zhaoyu Shi, Yuan Zhang, Jiaxin Yan, Fangfang Shang, Yao Wang, Huijian Lu, Hongmei Gu, Weiqiang Dou, Xinquan Wang, Li Yuan

**Affiliations:** ^1^Department of Nephrology, Affiliated Hospital of Nantong University, Jiangsu, China; ^2^Department of Ultrasound Medicine, The Second Affiliated Hospital of Nantong University, Jiangsu, China; ^3^Department of Medical Imaging, Affiliated Hospital of Nantong University, Jiangsu, China; ^4^MR Research, GE Healthcare, Beijing, China

**Keywords:** native T1 mapping, kidney fibrosis, chronic glomerulonephritis, kidney biopsy, magnetic resonance imaging, chronic kidney disease

## Abstract

**Purpose:** To assess the utility of non-contrast enhanced native T1 mapping of the renal cortex in assessing renal fibrosis for patients with chronic glomerulonephritis (CGN).

**Methods:** A total of 119 patients with CGN and 19 healthy volunteers (HVs) were recruited for this study. Among these patients, 43 had undergone kidney biopsy measurements. Clinical information and biopsy pathological scores were collected. According to the results of the renal biopsy, the patients were classified into the high (25–50%), low (<25%) and no renal interstitial fibrosis (IF) (0%) groups. The correlations between the T1 value in the renal cortex and each of the clinical parameters were separately analyzed. The relationships between each fibrosis group and the T1 value were also evaluated and compared between groups. Binary logistic regression analysis was further used to determine the relationship between the T1 value and renal fibrosis. Receiver operating characteristic (ROC) curves were plotted to analyze the diagnostic value of the T1 value for renal fibrosis.

**Results:** Compared with those of the HVs, the T1 values were significantly higher in patients at all stages of chronic kidney disease (CKD) (all *p* < 0.05). Significant T1 differences were also revealed between patients with different stages of CKD (*p* < 0.05). Additionally, the T1 value correlated well with CKD stage (*p* < 0.05), except between CKD 2 and 3. In addition, the T1 value was positively correlated with cystatin C, neutrophil gelatinase-associated lipocalin, and serum creatinine and negatively correlated with hemoglobin, kidney length, estimated glomerular filtration rate and hematocrit (all *p* < 0.05). Compared with those of the no IF group, the T1 values were increased in the low- and high-IF groups (both *p* < 0.05). Logistic regression analysis showed that an elevated T1 value was an independent risk factor for renal fibrosis. ROC analysis suggested that the optimal critical value of T1 for predicting renal fibrosis was 1,695 ms, with a specificity of 0.778 and a sensitivity of 0.625.

**Conclusion:** Native T1 mapping demonstrated good diagnostic performance in evaluating renal function and was an effective noninvasive method for detecting renal fibrosis in CGN patients.

## Introduction

Chronic kidney disease (CKD) has been increasingly recognized as a global public health problem (1). Currently, chronic glomerulonephritis (CGN) remains the leading cause of CKD and end-stage renal disease (ESRD) in China (2). Renal interstitial fibrosis is the abnormal deposition of collagen and associated proteins in the interstitium of the renal cortex. It is a common histological abnormality in all types of renal diseases and is considered to be a key predictor of renal functional recovery and prognosis in most renal diseases (3, 4). Currently, kidney biopsy is the gold standard for fibrosis evaluation; however, due to its shortcomings, such as its invasiveness and low reproducibility and the limited size of the collected sample, this method has not been widely implemented in the clinic (5). Therefore, there is a great clinical need for a method for noninvasively evaluating the degree of renal fibrosis.

T1 mapping is a quantitative magnetic resonance imaging (MRI) technique that has been reported to reflect the degree of tissue fibrosis and thus might serve as an alternative approach to kidney biopsy (6). T1 mapping has been widely used in the quantitative assessment of diffuse myocardial fibrosis and in evaluating the degree and staging of liver fibrosis (7, 8).

Previously reported techniques for T1 mapping acquisition were developed based on a variety of mathematical models, including the modified looker-locker inversion-recovery (MOLLI) and the variable flip angle model (6, 9). While good results have been obtained, apparent but not native T1 or less reproducible T1 values were reported using these methods. In comparison, the so-called saturation method using adaptive recovery times (SMART) technique [using single-point, saturation-recovery fast imaging employing steady-state acquisition (FIESTA)] has been proposed for native T1 acquisition with high accuracy and repeatability and low variability (10). However, the SMART technique for native T1 mapping is still limited in the grading of kidney fibrosis for CGN patients. Therefore, the main goal of this study was to investigate the feasibility of native T1 mapping with the SMART technique in assessing renal function and kidney fibrosis in CGN patients.

## Materials and Methods

### Subject Recruitment

This study was conducted with prior approval from the Ethics Committee of the Affiliated Hospital of Nantong University (2019-K070). Nineteen healthy volunteers (HVs) were selected as normal controls, and a total of 119 patients with CGN who were hospitalized in the Department of Nephrology, Affiliated Hospital of Nantong University from September 2019 to August 2020 were enrolled in the CKD group.

The inclusion criterion for CGN patients was a history of proteinuria or/and glomerular hematuria for more than 3 months, excluding secondary glomerulonephritis and hereditary glomerulonephritis. The CKD stage criteria were followed according to the Kidney Disease Outcomes Quality Initiative (K/DOQI) guidelines (11).

All subjects underwent breath-holding training and fasting for at least 6 h prior to MRI acquisition and signed informed consent forms. Each MRI measurement was performed within 1 week prior to renal biopsy analysis if performed.

### Clinical Parameters

Clinical data and laboratory test results were collected for all patients. Clinical data included age, sex, blood pressure and body mass index (BMI), and the laboratory examinations included 24-h urinary protein (24-h UP), albumin (Alb), hemoglobin (Hb), hematocrit (Hct), renal length diameter (via B-ultrasound), neutrophil gelatinase-associated lipocalin (NGAL), cystatin C (CysC), and serum creatinine (SCr). The estimated glomerular filtration rate (eGFR) was calculated according to the Chronic Kidney Disease Epidemiology Collaboration (CKD-EPI) formula (12).

### Magnetic Resonance Imaging

SMART T1 MRI examinations were performed for each subject in the coronal view. Nine different inversion recovery (IR) times of 100, 120, 140, 160, 998, 1,855, 2,748, 3,568, and 2,000 ms were used for T1 map calculation. The applied scan parameters were slice thickness = 5 mm, spacing = 1 mm, number of slices = 10, field of view ranging from 30 × 30 cm to 36 × 36 cm, matrix = 192 × 128, number of excitations (NEX) = 1 and acceleration factor = 2. A respiration trigger was also adopted. The scan time was 3 min.

Using vendor-provided postprocessing software embedded in a GE advanced workstation (ADW4.6), coronal renal T1 maps were acquired accordingly for each subject. On renal T1 maps, three regions of interest (ROIs) were manually drawn on the upper, middle, and lower parts of each renal cortex by a senior radiologist. The corresponding T1 values of the three renal subregions were obtained for statistical analysis.

### Renal Pathological Analysis

Formalin-fixed renal tissue was embedded in paraffin. Two-micrometer paraffin sections were cut and stained with Masson and periodic acid-silver methenamine (PASM). The Katafuchi semiquantitative scoring system (13) was used to score the degree of pathological injury. According to the renal pathology, the degree of renal interstitial fibrosis (IF) in this study was graded from 0 to 50%; therefore, renal IF was further evaluated and classified into high (25–50%), low (<25%) and no IF groups (0%).

### Statistical Analyses

Data were analyzed using IBM SPSS Statistics version 25.0 (IBM, Armonk, New York, U.S.A.). Differences in T1 values between the CKD and HV groups were assessed using one-way analysis of variance (ANOVA) with Bonferroni correction for multiple comparisons. Analysis of covariance (ANCOVA) was used to adjust for the effects of clinically relevant differences in baseline characteristics between different groups. Differences in T1 values between groups with different degrees of fibrosis were assessed using the Wilcoxon rank-sum test. Pearson or Spearman correlation coefficients (normality test-dependent) were used to assess the relationship between the T1 value and clinical indexes or between the T1 value and pathological score. Univariate and multivariate logistic regression analyses were utilized to determine the relevant risk factors for renal fibrosis. Receiver operating characteristic (ROC) curve analysis was used to evaluate the efficacy of T1 in the diagnosis of renal fibrosis. A *p* < 0.05 was considered statistically significant.

## Results

A total of 19 HVs and 119 CKD patients (CKD 1–5: 33, 26, 25, 16, and 19, sequentially) were enrolled in this study. Among these patients, 43 underwent renal biopsy (CKD 1–3: 20, 14, and 9). Neither the HVs nor the CKD patients had diabetes mellitus. Significant differences were found in systolic blood pressure (SBP), 24-h UP, CysC, NGAL, Scr, Hb, renal length, eGFR, and Hct between pairs of CKD subgroups. The demographics of the CKD subgroups are shown in Table 1.

**Table 1:** The demographics and Clinical parameters of CKD sub-groups.

	**CKD1 (*n* = 33)**	**CKD2 (*n* = 26)**	**CKD3 (*n* = 25)**	**CKD4 (*n* = 16)**	**CKD5 (*n* = 19)**	**F/χ^2^**	***P***
Age (years)	43 (32,55)	46 (29,53)	50 (44,64)	52 (39,62)	53 (32,66)	7.552	0.109
Males (%)	19 (58)	18 (69)	18 (72)	8 (50)	12 (63)	2.879	0.523
BMI (Kg/m^2^)	24.3 (20.95,26.25)	23.70 (22.80,25.10)	24.60 (22.35,26.60)	24.50 (21.70,27.60)	24.20 (19.40,26.40)	1.081	0.897
SBP (mmHg)	135 (130,142)	136 (133,143)	135 (128,150)	143 (132,155)	152 (135,163)	9.881	0.042
DBP (mmHg)	85.03 ± 8.81	82.27 ± 11.63	81.44 ± 13.62	85.94 ± 14.71	86.53 ± 13.05	0.785	0.537
eGFR (ml/min/1.73 m^2^)	104.42 (94.82,115.71)	75.20 (65.25,81.29)	48.54 (40.50,51.61)	22.40 (19.92,24.95)	7.96 (6.57,11.00)	112.4	<0.001
NGAL (ng/ml)	209.64 (134.30,280.03)	179.00 (156.56,206.63)	258.00 (182.15,337.22)	464.00 (342.30,523.82)	506.00 (450.10,609.60)	54.838	<0.001
CysC (mg/L)	0.90 (0.79,1.03)	1.11 (0.97,1.23)	1.48 (1.32,1.71)	2.20 (1.99,2.51)	3.30 (3.09,3.84)	96.911	<0.001
SCr (μmol/L)	64 (56,78)	95.5 (86,108.25)	132 (123,156)	253 (230,312.50)	609 (459,748)	107.216	<0.001
Alb (g/L)	29.6 (23.15,36.80)	35.25 (22.23,41.34)	36.5 (33.55,41.55)	36.50 (30.08,41.08)	36.6 (31.30,40.40)	8.644	0.071
24-h UP (g)	2.57 (1.04,5.19)	1.59 (0.83,4.02)	2.00 (0.91,3.92)	1.67 (1.15,4.41)	4.00 (1.14,6.22)	3.693	0.449
Hb (g/L)	130.52 ± 21.39	138.23 ± 22.89	127.16 ± 23.16	117.81 ± 23.45	87.26 ± 25.99	15.423	<0.001
Hct (L/L)	0.38 ± 0.06	0.41 ± 0.06	0.38 ± 0.07	0.35 ± 0.07	0.26 ± 0.07	14.829	<0.001
Renal length (mm)	109 (103,116)	105 (101,110)	97 (91.50,110)	94 (90.25,99.75)	89 (85,95)	47.904	<0.001

*BMI, Body mass index; SBP, Systolic blood pressure; DBP, Diastolic blood pressure; Alb, albumin; 24-h UP, 24-h urinary protein; Hb, hemoglobin; Hct, hematocrit*.

### T1 Analysis for HVs and CKD Subgroups

**Figure 1:**
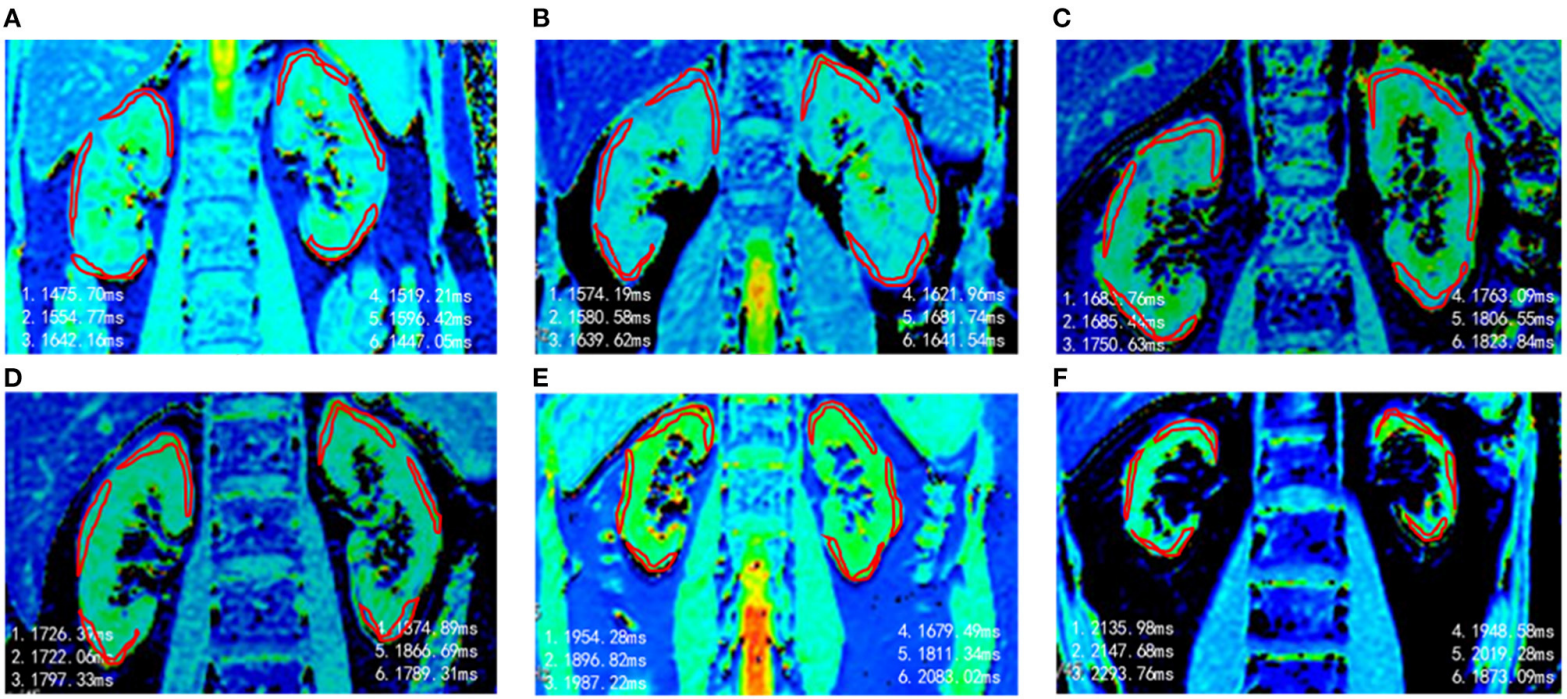
Representative T1 maps of renal cortex in different groups. **(A)** HVs, T1 = 1,539 ms. **(B)** CKD 1, T1 = 1,622 ms. **(C)** CKD 2, T1 = 1,751 ms. **(D)** CKD 3, T1 = 1,796 ms. **(E)** CKD4, T1 = 1,902 ms. **(F)** CKD 5, T1 = 2.068 ms.

**Figure 2:**
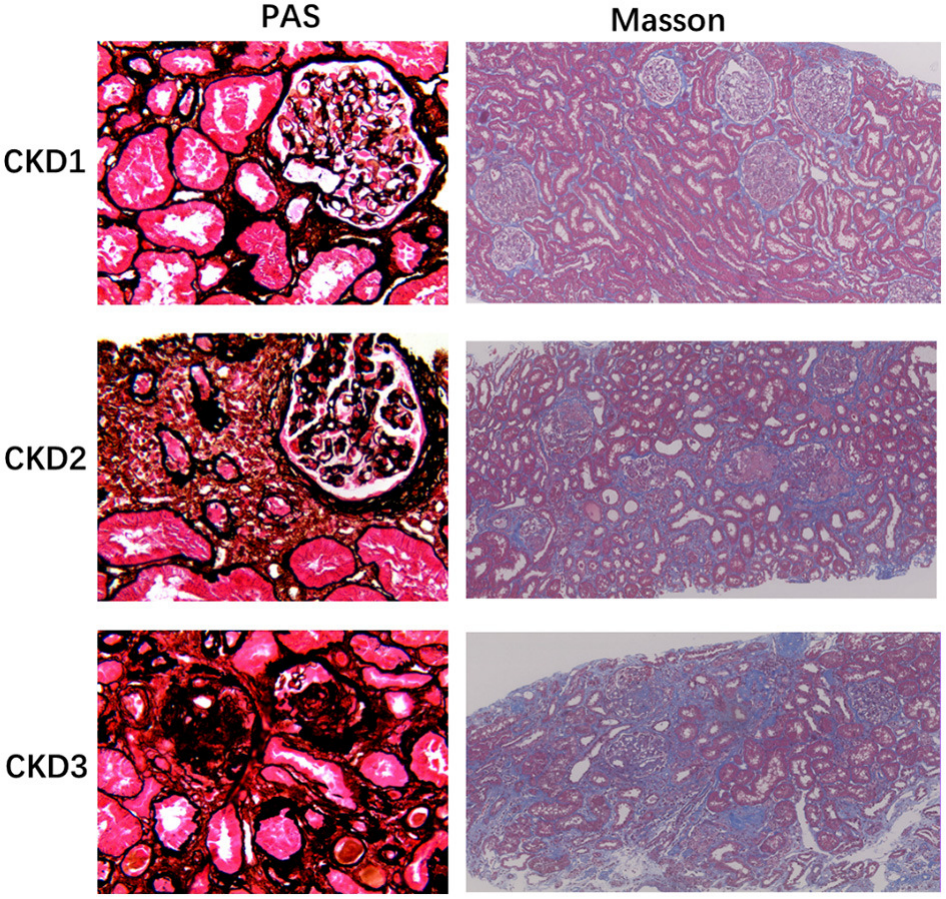
Histology of chronic glomerulonephritis of CKD1-3. PASM stained sections of the renal cortex show progressive glomerular changes, such as glomerulosclerosis (40×). Masson-stained section reveals progressive tubulointerstitial fibrosis, as no, low, and high IF, respectively (10×).

Representative renal T1 images of the HV and CKD 1–5 patients and the pathological results for renal biopsy patients (CKD 1–3) are shown in Figures 1, 2.

**Figure 3:**
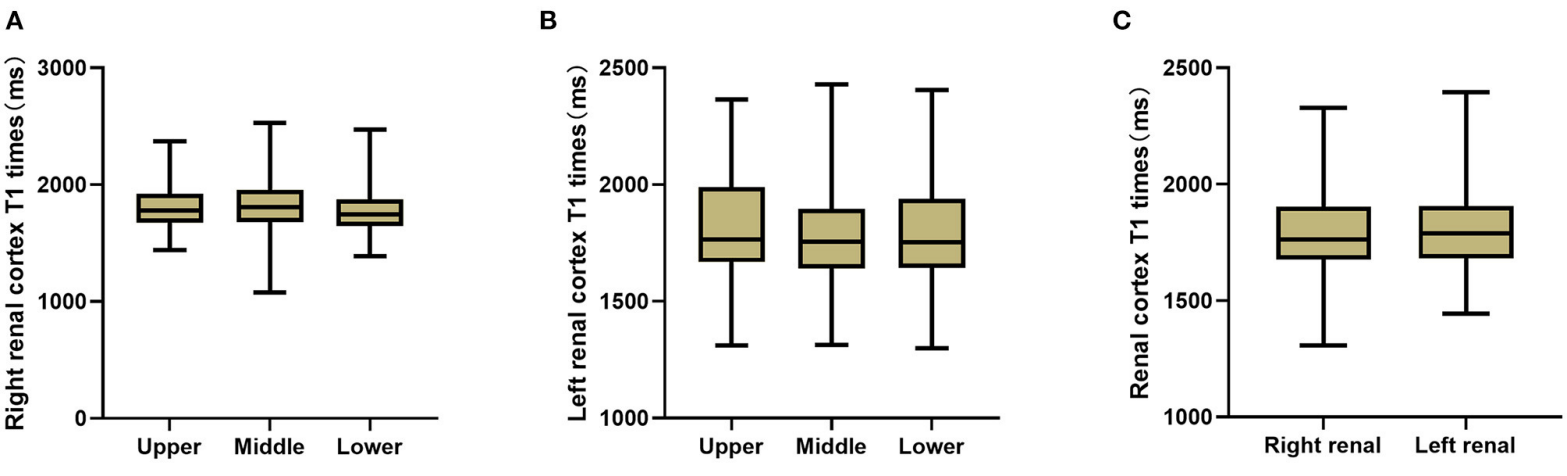
T1 comparison between each two sub-regions of right kidney **(A)** and left kidney **(B)**, and between bilateral kidneys **(C)**. No significant T1 difference of the renal cortex was found (*P* > 0.05).

For each subject, one-way ANOVA was used to assess the differences in the T1 value among the three (upper, middle and lower) subregions of two kidneys; however, the differences were not significant (left renal: *F* = 2.024, *P* > 0.05, right renal: *F* = 1.353, *P* > 0.05), so the average T1 values of the three poles of each kidney were taken as their own values. The paired sample t test was further applied to compare the T1 values between the two kidneys, but again, no significant difference was found (*P* > 0.05). Therefore, the mean T1 value of the bilateral kidneys was used to represent the T1 value of the renal cortex (Figure 3).

**Figure 4:**
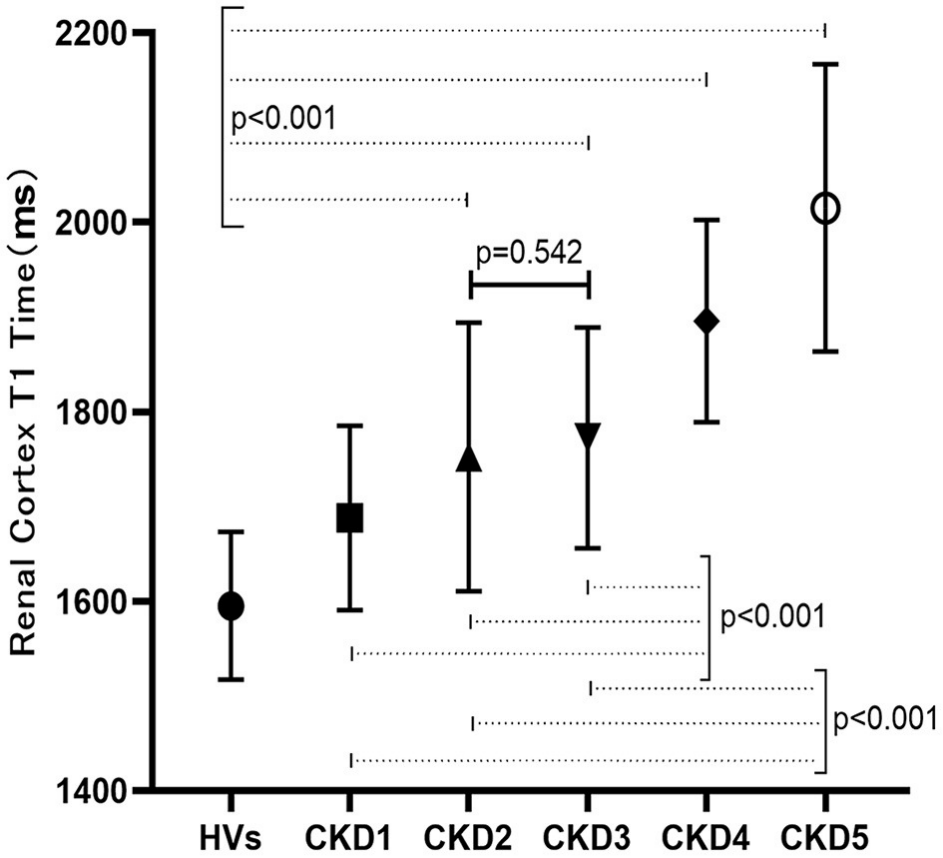
T1 Comparison among HVs and CKD sub-groups.

Using ANCOVA analysis, different hemoglobin levels were adjusted for each group, and significantly different T1 values were found among the HVs and each CKD subgroup (*F* = 29.62, *p* < 0.001). Multiple comparative analyzes were performed and showed that significantly different renal T1 values were present between any two groups except between CKD 2 and 3 (*P* > 0.05; Figure 4).

### Correlation Analysis Between T1 Value and Clinical Indexes

**Figure 5:**
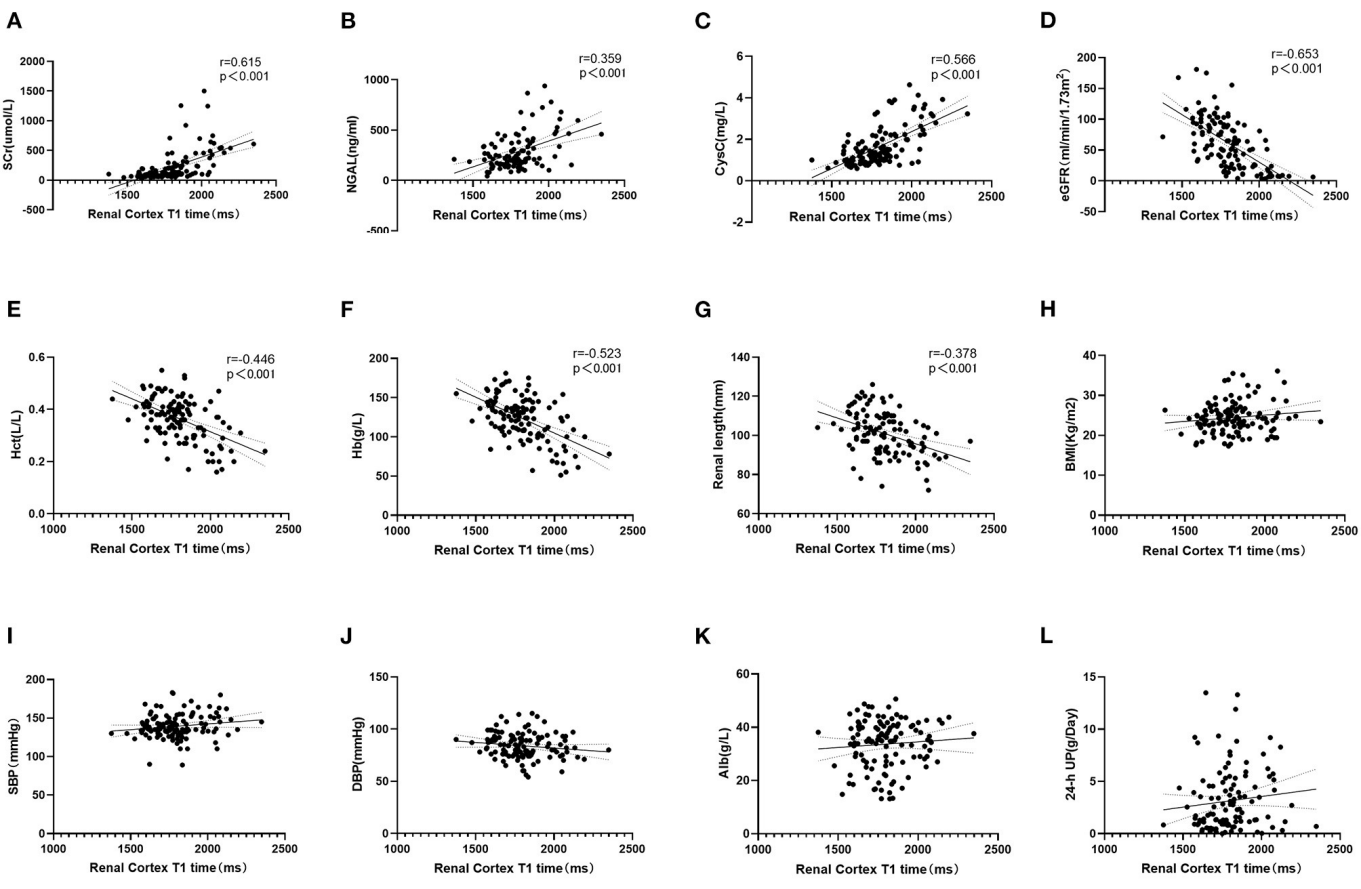
**(A–C)** Correlation analysis between T1 and clinical indexes. T1 value was positively correlated with SCr, NGAL and CysC (*p* < 0.001). **(D–G)** Negatively correlated with eGFR, Hct, Hb, and Renal length (*p* < 0.001). **(H–L)** T1 value had no correlation with BMI, SBP, DBP, Alb, and 24-h UP (*p* > 0.05).

Neither Pearson or Spearman correlation analysis revealed a correlation between T1 and BMI, diastolic blood pressure (DBP), SBP, Alb, or 24-h UP (*P* > 0.05). However, the T1 value was positively correlated with CysC, NGAL and SCr (*p* < 0.001, *r* = 0.566, 0.359, 0.615, respectively) and negatively correlated with Hb, renal length, eGFR and Hct (*p* < 0.001, *r* = 0.523, 0.378, 0.653, 0.446, respectively; Figure 5).

### Data Analysis Between T1 Values and Pathological Findings

**Figure 6:**
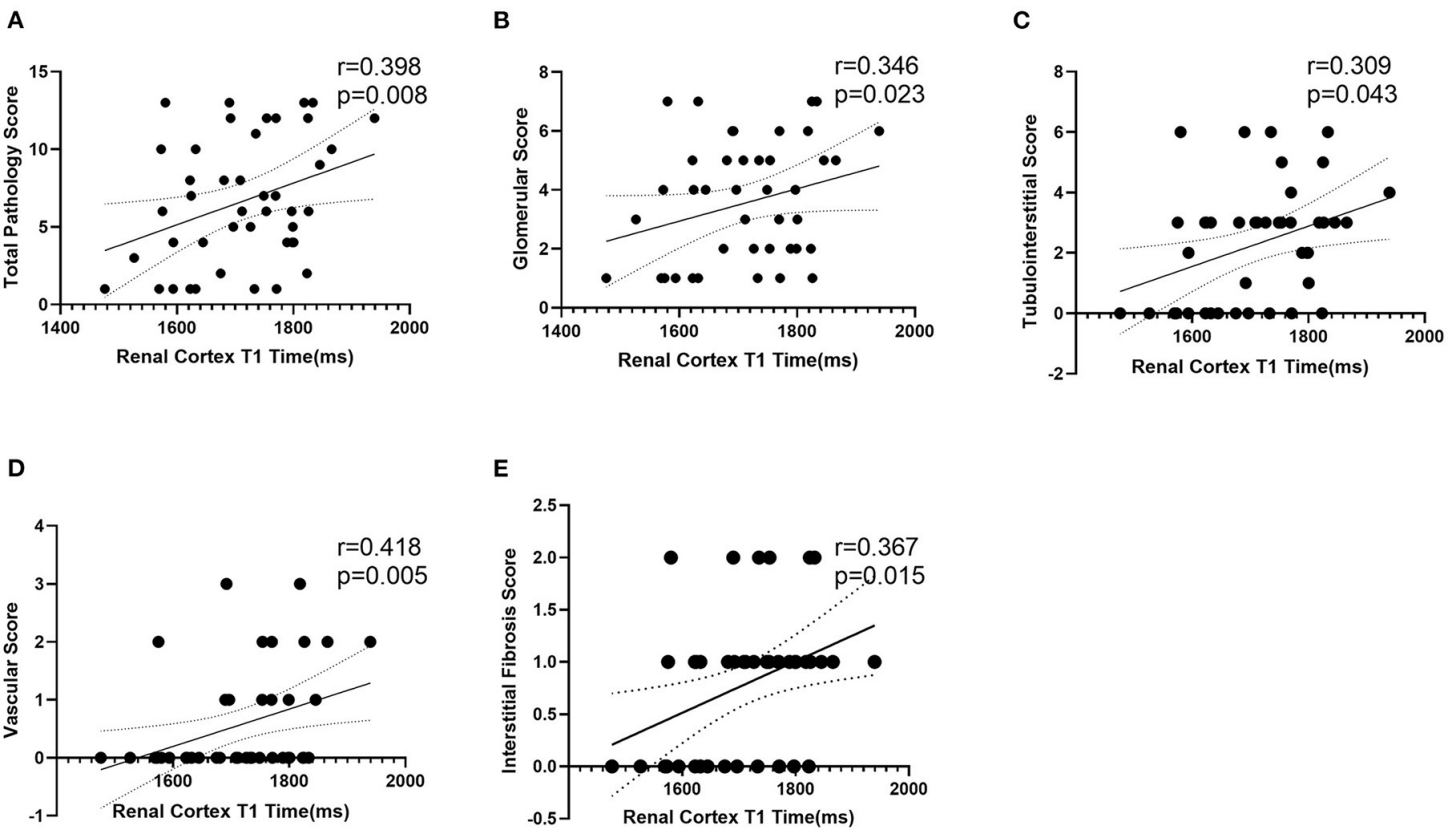
Correlation analysis between T1 and pathological score. T1 value of renal cortex was positively correlated with total pathological **(A)**, glomerular **(B)**, tubulointerstitial **(C)**, vascular **(D)**, and interstitial fibrosis **(E)** score (*p* < 0.05).

**Figure 7:**
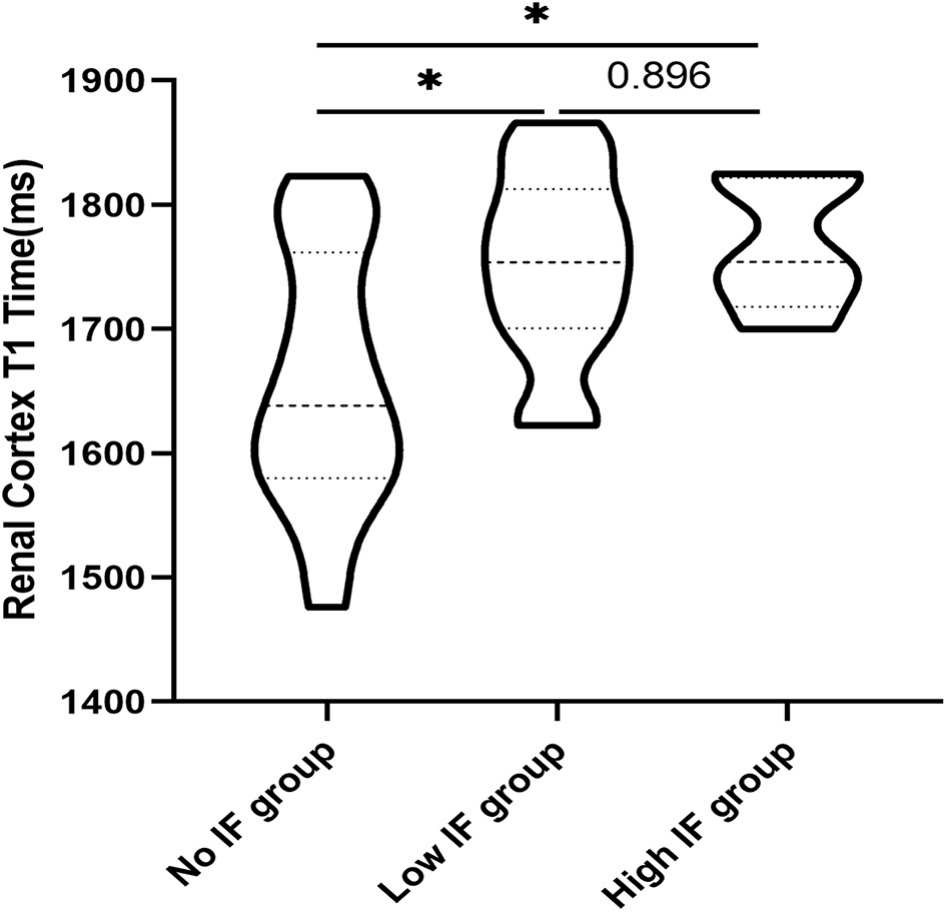
Comparison of T1 values in the renal cortex of patients with different degrees of IF. T1 values in the renal cortex of the no IF group were lower than those of the low and high fibrosis groups (*p < 0.05), while T1 values in the renal cortex of the low and high fibrosis group had no significant difference (*P* > 0.05).

One-way ANOVA showed that T1 was positively correlated with the total pathological, glomerular, tubulointerstitial, vascular and interstitial fibrosis scores (*p* < 0.05; Figure 6). In addition, according to the pathological findings, three IF groups (no, low, and high) were formed. A lower renal cortex T1 value was found in the no IF group than in the low- and high-IF groups (1,658 ± 104 ms vs. 1,752 ± 75 ms vs. 1,767 ± 54 ms, both *p* < 0.05), while comparable T1 values were shown between the low- and high-IF groups (*P* > 0.05; Figure 7).

### ROC Analysis of the T1 Value in Predicting Renal Fibrosis

**Figure 8:**
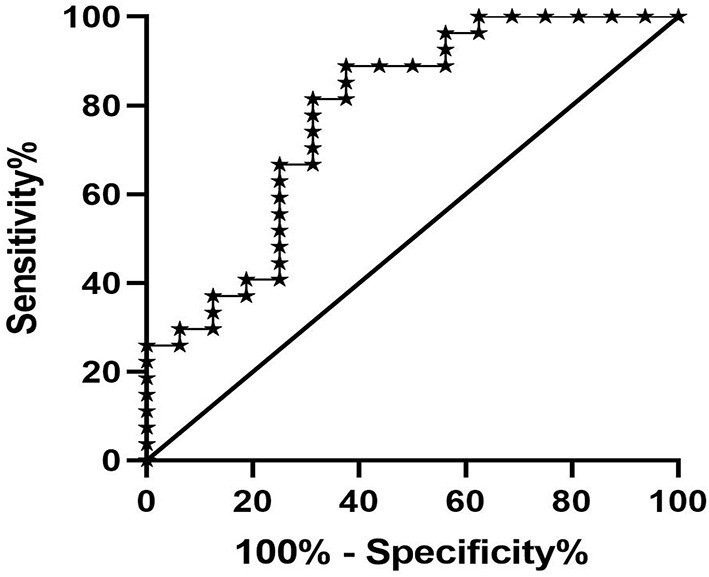
ROC analysis of T1 value in predicting renal fibrosis. The area under the ROC curve was 0.762, the optimal cut-off value was 1,695 ms, the specificity was 0.778, and the sensitivity was 0.625.

Univariate logistic regression analysis indicated that T1 value, eGFR, SCr, CysC, and Alb were the main influencing factors for renal fibrosis. Multivariate logistic regression analysis further suggested that the T1 value could independently predict the occurrence of renal fibrosis (Table 2). ROC curve analysis was then applied to evaluate the efficacy of T1 in predicting renal fibrosis. The resultant area under the ROC curve was 0.762 (95% CI, 0.609–0.914, *p* < 0.05) with an optimal critical value of 1,695 ms, a specificity of 0.778, and a sensitivity of 0.625, suggesting that the occurrence of renal fibrosis should be suspected at renal cortex T1 values of higher than 1,695 ms (Figure 8).

**Table 2:** Influencing factors of renal fibrosis (Logistic regression analysis).

	**Univariate analysis**		**Multivariate analysis**	
	**OR (95% CI)**	**P值**	**OR (95% CI)**	**P值**
Age (years)	0.968 (0.922~1.016)	0.185		
T1 (ms)	1.011 (1.003~1.019)	0.005	1.017 (1.003~1.031)	0.020
BMI (Kg/m^2^)	1.107 (0.931~1.318)	0.250		
SB (mmHg)	1.008 (0.968~1.050)	0.691		
DB (mmHg)	0.996 (0.936~1.058)	0.887		
eGFR (ml/min/1.73 m^2^)	0.961 (0.932~0.991)	0.011	1.023 (0.963~1.086)	0.461
NGAL (ng/ml)	1.005 (0.996~1.015)	0.264		
CysC (mg/L)	34.326 (1.202~980.070)	0.039	0.038 (0.000~104.836)	0.418
Scr (μmol/L)	1.056 (1.017~1.096)	0.005	1.100 (0.987~1.277)	0.086
Alb (g/L)	1.102 (1.018~1.192)	0.016	1.059 (0.947~1.185)	0.314
24-h PU (g)	0.841 (0.668~1.058)	0.139		
Hb (g/L)	1.002 (0.972~1.033)	0.913		
Hct (L/L)	0.029 (0.000~838.105)	0.499		
Renal length (mm)	0.946 (0.867~1.032)	0.208		

## Discussion

Although T1 mapping is a promising method for assessing and monitoring fibrosis noninvasively, only a few studies have investigated its value in the assessment of renal fibrosis. In the present study, we demonstrated that renal cortex T1 values reflect the level of renal function and the degree of histopathological changes.

Previous studies (6, 14) have shown that compared with that of the control group, the T1 value of the renal cortex in CKD patients is increased and the differentiation of the cutaneous medulla is significantly decreased. As we all know, methods to measure GFR would be classic to estimation of kidney function but laborious, expensive, and not broadly available (15). In our study, we used the CKD-EPI formula to estimate GFR, which is widely available and appropriate for use as a first line tool. We also found an increased T1 value with higher CKD stage, indicating a good correlation between T1 and renal function. There was a significant difference in renal T1 values between each pair of patient groups except between the CKD 2 and 3 groups, indicating that the T1 parameter can be used in the staging of CKD. No significant difference in the T1 value was found between the CKD 2 and 3 patient, which might be explained by the subtle fibrosis differences between the two groups or the limited number of patients enrolled. Compared with that of HVs, the T1 value increased even in CKD stage 1 (1,609 ± 99 ms vs. 1,688 ± 97 ms, *p* < 0.05). Therefore, T1 mapping may be able to sensitively evaluate early renal function and pathological impairment in patients with CGN.

Almost all cells are involved in the complex process of renal fibrosis, including fibroblasts, renal tubular epithelial cells, endothelial cells, vascular smooth muscle cells, mesangial cells, Sertoli cells and infiltrating cells (16). A decrease in circulating blood flow and oxygen supply as well as changes in renal hardness will change the T1 value. T1 mapping has been used to satisfactorily estimate fibrosis in CKD mice (17). Among previous studies that have conducted renal T1 mapping in patients with renal disease, only a few have observed correlations between the T1 value and renal pathology (9, 18, 19). Friedli et al. (18) found that the T1 value had a good correlation with the degree of both fibrosis and inflammation and could thus be used to evaluate the degree of renal interstitial fibrosis in transplanted kidneys. In IgA nephropathy patients, Graham-Brown et al. (9) found that the T1 value tended to increase in patients with high interstitial damage score.

In our study, CGN patients who underwent renal biopsy had renal function at CKD stages 1 to 3, and the Katafuchi semiquantitative scoring system was used to quantify pathological changes. The T1 value of the renal cortex was positively correlated with the glomerular, tubular, vascular and renal fibrosis scores, indicating the efficacy of noninvasive T1 mapping in evaluating renal pathology. Furthermore, we found that compared with the no IF group, the low (<25%) and high (25–50%) IF groups showed significantly increased T1 values, suggesting that the T1 parameter is a sensitive indicator of renal IF. In addition, the T1 value in the high IF group was higher than that in the low IF group, although the difference was not significant. Buchanan et al. (19) identified significant differences between “Low” and “High” interstitial fibrosis at 40% in the cortical T1 value in CKD 3–4 patients. The differences in the T1 value in the discrimination of renal fibrosis may be related to the severity of renal lesions in the CKD patients included in the two studies. A large clinical cohort should be analyzed to further validate the clinical value of T1 in the evaluation of renal fibrosis in a follow-up study.

Additionally, in this study, the T1 value was positively correlated with SCr, NGAL and CysC and negatively correlated with Hb, renal length, eGFR and Hct. A variety of indirect indicators are used clinically to preliminarily evaluate renal fibrosis. For example, SCr, CysC, and eGFR are commonly used indicators to evaluate glomerular filtration function (20). NGAL can reflect renal tubular function as an early marker of CKD, and its level is correlated with CKD stage (21). Patients with CKD have shown significant increases in the occurrence and severity of anemia (22), whose degree can be reflected in Hb and Hct. CKD patients have different degrees of glomerulosclerosis, renal tubule atrophy and interstitial fibrosis, resulting in renal shrinkage, and renal size is well correlated with renal function (23). Therefore, our findings indicate a good correlation between the T1 value and traditional clinical indirect indicators for the evaluation of renal fibrosis.

There are also some limitations to this study. First, as a single-center study, the number of renal biopsy patients in the cohort was relatively small. Second, all CGN patients who underwent renal biopsy had CKD stages 1–3, and no patients had more than 50% renal IF. Third, this study only focused on CGN; more research is required to investigate the clinical value of T1 mapping in other renal diseases.

## Conclusion

In summary, this study found that the renal cortex T1 value was significantly increased in CGN patients and was well correlated with CKD stage, renal fibrosis and renal function indicators. With these promising findings, T1 mapping has demonstrated good diagnostic performance in the evaluation of renal function and the noninvasive detection of CGN fibrosis.

## Data Availability Statement

The original contributions presented in the study are included in the article/supplementary material, further inquiries can be directed to the corresponding authors.

## Ethics Statement

The studies involving human participants were reviewed and approved by the Ethics Committee of the Affiliated Hospital of Nantong University (2019-K070). The patients/participants provided their written informed consent to participate in this study.

## Author Contributions

LY is the guarantor of integrity of entire study and approved this manuscript finally. LY and XW designed this study. YZ, JY, FS, YW, HL, and HG did the MR scan and finished data acquisition. JW and ZS analyzed the data, conducted statistical analysis, and prepare manuscript. JW, ZS, WD, and LY edited and revised the manuscript. All authors contributed to the article and approved the submitted version.

## Funding

This work was funded by the Jiangsu Province TCM science and technology development plan project (grant no. YB201985), Clinical and Experimental Research of YSHS Granule, National Key Research and Development Project (grant no. 2019YFC1709402), and Nantong Science and technology project (grant no. MS12020042).

## Conflict of Interest

WD was employed by GE Healthcare. The remaining authors declare that the research was conducted in the absence of any commercial or financial relationships that could be construed as a potential conflict of interest.

## Publisher's Note

All claims expressed in this article are solely those of the authors and do not necessarily represent those of their affiliated organizations, or those of the publisher, the editors and the reviewers. Any product that may be evaluated in this article, or claim that may be made by its manufacturer, is not guaranteed or endorsed by the publisher.
